# Real-World Safety Concerns of Tirzepatide: A Retrospective Analysis of FAERS Data (2022–2025)

**DOI:** 10.3390/healthcare13182259

**Published:** 2025-09-09

**Authors:** Hadi A. Almansour, Hilal A. Thaibah, Moaddey Alfarhan, Saeed A. Al-Qahtani, Amani A. Khardali, Thamir M. Alshammari

**Affiliations:** 1 Department of Clinical Practice, College of Pharmacy, Jazan University, Jazan 45142, Saudi Arabia; halmansour@jazanu.edu.sa (H.A.A.);; 2 Pharmacy Practice Research Unit, College of Pharmacy, Jazan University, Jazan 45142, Saudi Arabia

**Keywords:** adverse drug events, FAERS, GLP-1 agonists, medication errors, Mounjaro, pharmacovigilance, tirzepatide, Zepbound

## Abstract

**Background**: Tirzepatide (Mounjaro or Zepbound), a dual GLP-1/GIP receptor agonist, is approved for type 2 diabetes and weight management. Despite its efficacy, real-world safety data remain limited. This study analyzed post-marketing adverse events (AEs) associated with tirzepatide using the FDA Adverse Event Reporting System (FAERS) to identify emerging safety concerns. **Methods**: FAERS reports from 2022 to Q1 2025 were analyzed. Disproportionality analyses (proportional reporting ratio [PRR], reporting odds ratio [ROR], empirical Bayes geometric mean [EBGM], and information component [IC]) were performed to detect safety signals. Reports were stratified by year, demographics, and AE type, focusing on cases in which tirzepatide was the primary suspect. **Results**: Among 65,974 reports, the majority originated from the U.S. (96%), with middle-aged females (40–59 years; 67%) most frequently affected. Incorrect dose administration was the top AE, increasing 8-fold from 1248 (2022) to 9800 (2024), with strong risk signals (ROR 22.15, 95% CI (20.75–23.65), and ROR 23.43, 95% CI (22.82–24.05), respectively, and PRR 16.80, 95% CI (15.74–17.93), and PRR 17.62, 95% CI (17.16–18.09), respectively). Other common AEs included injection-site reactions (e.g., pain [5273 cases in 2024]), gastrointestinal issues (nausea [3602 in 2024]), and off-label use. Class-related AEs (e.g., decreased appetite and blood glucose fluctuations) were also reported. **Conclusions**: Tirzepatide is associated with significant dosing errors, injection-site reactions, and gastrointestinal AEs in real-world use. The rising trend in reports underscores the need for enhanced provider and patient education, clearer dosing guidelines, and proactive monitoring. Further research is warranted to explore causative factors and optimize risk mitigation strategies.

## 1. Introduction

Tirzepatide (Mounjaro or Zepbound) is a medication used for managing type 2 diabetes, after it was approved by the FDA in May 2022, and, more recently (November 2023), for weight management [1,2]. It is a glucagon-like peptide-1 (GLP-1) receptor agonist used to regulate blood glucose levels [2]. Clinical trials have demonstrated that tirzepatide can lead to substantial weight loss in individuals with and without diabetes [3]. However, long-term safety data on it for this indication are still being collected [3,4]. There have been many social media advertisements and influencers promoting the use of tirzepatide for weight loss [5]. It has attracted attention both for its effectiveness and for possible side effects [5]. Thus, the safety of tirzepatide needs to be understood by healthcare providers and patients.

Medication errors remain a major concern in healthcare, affecting patient safety, treatment efficacy, and healthcare cost [6,7]. Since medication errors can cause avoidable adverse drug events (ADEs), it is important to know the factors that cause such medication errors to enhance patient safety [7]. Medication errors are possible at all phases of the medication therapy process, including prescribing, transcribing, dispensing, administering, and monitoring, and at all levels of these processes [8]. Such errors may result in ADEs, which are frequently avoidable. The American Society of Health-System Pharmacists (ASHP) has worked to prevent these mistakes, with a principal focus on system-level changes in, as well to improve, hospitals [9]. In a systematic review, Assiri et al. (2018) defined the epidemiology of medication errors and types of common errors and risk factors at the level of the community-care environment [10]. It was indicated that there is a need for better reporting and monitoring of errors to better grasp the size of the problem.

There are reports of medication errors and safety concerns in the Food and Drug Administration’s (FDA) Adverse Event Reporting System (FAERS) database. The FAERS is a system that aids the FDA’s post-marketing safety surveillance program for all marketed drugs and therapeutic biologic products [11]. It includes adverse event reports that are provided by manufacturers as required by regulation, in addition to reports provided directly by healthcare providers and consumers/patients. Using the FAERS data, investigators can identify and describe adverse events (AEs) unique to tirzepatide to build its safety profile in relation to other drugs sharing common side effect profiles.

Several studies have underlined the importance of discovering drug combinations that mitigate ADEs [12,13]. Since tirzepatide can potentially be used in combination with several adjunctive diabetes therapies, knowledge of possible drug interactions is relevant [12]. This could suggest that analysis of FAERS data on tirzepatide may provide insights into interactions that either worsen or mitigate ADEs [14] and, therefore, have implications for developing clinical guidelines for safer prescribing practices. Although there are barriers that may impact medication error reporting, the resolution of these barriers is critical for the appropriate characterization of the safety record of tirzepatide in the FAERS database, in turn, paving the way for more stringent policies to protect patients [15,16].

Pharmacological safety is an absolute concern in clinical settings. Various research studies describe the growing adoption of herbal medicines, and the issue of how adverse reactions are monitored demonstrates the need for strong methodologies to evaluate them [17]. This is consistent in many studies, which report a substantial prevalence of underreporting of AEs for novel drugs, such as tirzepatide [18]. Development of integrated surveillance systems is urgent, and especially relevant for drugs with new mechanisms of action like tirzepatide. Thus, the aim of this study is to provide an overview of the safety profile of tirzepatide, including medication errors, ADEs, and other safety issues using the FAERS database.

## 2. Methods

The study utilized data from the FAERS, covering reports from 2022 to 2025. Reports are submitted by various sources globally, including healthcare professionals, pharmaceutical manufacturers, lawyers, and patients. This database is freely accessible on the FDA’s website and is updated every three months [19]. The FAERS database is structured into the following seven distinct file types: Demographic and Administrative Information (DEMO), Adverse Events (REAC), Patient Outcomes (OUTC), Report Sources (RPSR), Drug Information (DRUG), Indications for Use (INDI), and Start and End Dates for Reported Drugs (THER). These sub-databases are classified based on the reporter type, including consumers, physicians, pharmacists, other healthcare professionals, or lawyers. Reported patient outcomes include death, hospitalization, disability, or other unspecified outcomes, all of which require intervention to be prevented. The country of the reporters indicates the basis of the latest report version, which can be inside or outside the U.S. Patient age is recorded numerically at the time of the event [19,20]. To link all datasets for the study, a unique identifier called “primaryid” was used. It is important to note that the U.S. FDA requires pharmaceutical manufacturers to report adverse drug reactions (ADRs) for their products. Consumers/patients and healthcare professionals are also recommended to submit ADR reports, despite their location [21,22].

The data retrieved from the FAERS for this study, from 2022 to the first quarter of 2025, are meant to demonstrate the safety concerns of tirzepatide use. The FAERS database uses the Medical Dictionary for Regulatory Activities’ (MedDRA) coding system, which employs preferred terms (PTs) to categorize AEs. The study was conducted in two phases to (1) find all AEs related to the drug of interest and (2) perform a disproportionality analysis on the most reported AEs. Reports were retrieved using the “drugname” and “prod_ai” variables. To minimize bias from other medications, only reports where tirzepatide was listed as the primary suspected (PS) cause of the adverse event were included. In addition, duplicate reports were identified and removed using the “primaryid”, event date (event_dt), and “pt” variables.

A non-case disproportionate/pharmacovigilance case analysis was performed to assess the risk of the most reported AEs linked to tirzepatide. In this analysis, “cases” were defined as potential AE reports associated with tirzepatide, while “non-cases” included all other AE reports for tirzepatide.

Both Bayesian and traditional statistical methods were used to evaluate possible safety concerns. A 2 × 2 contingency table was utilized to analyze the following four key metrics: “a” (cases for the studied medication), “b” (non-cases for the studied medication), “c” (cases for other medications), and “d” (non-cases for other medications) (Table 1). A dual-method approach was used to improve the reliability of the analysis and decrease the chance of inaccurate results. The disproportionality metrics calculated included the proportional reporting ratio (PRR), reporting odds ratio (ROR), information component (IC), and empirical Bayes geometric mean (EBGM). A safety signal was indicated if the results met specific criteria across the following metrics: an ROR above one, a PRR of two or higher, an EBGM exceeding two, or an IC value greater than zero [22,23].

To ensure reliability and minimize false associations, significant signals were required to meet the criteria for all four metrics. All statistical analyses were conducted using R software (version 4.2.2) and R Studio (version 2024.04.2+764).

## 3. Results

A total of 65,974 tirzepatide reports were retrieved from the U.S. FAERS database (Table 2). The majority of reports were submitted by females (44,120; 66.9%), followed by males (13,410; 20.3%), while 8444 (12.8%) were by an unspecified sex. Regarding age, the highest number of reports came from individuals aged 40 to 59 years (21,809; 33.1%), followed by those aged 60 to 89 years (12,943; 19.6%) and those aged 18 to 39 years (7519; 11.4%). Reports involving children under 18 were infrequent (16; 0.02%). Notably, 23,666 reports (35.9%) did not specify age.

Regarding reporter types, consumers (61,838; 93.7%) were the predominant source of reports. Among healthcare professionals, medical doctors submitted 1110 (1.7%) reports, pharmacists submitted 691 (1.0%), and other healthcare professionals contributed 2062 (3.1%). A smaller portion of reports originated from lawyers (222; 0.3%), and 51 (0.1%) were from other or unknown sources.

The most frequently reported outcomes were categorized as unknown (59,171; 89.7%). Among the known outcomes, hospitalization (2418; 3.7%) was the most common, followed by other outcomes (3983; 6.0%), death (221; 0.3%), and disability (181; 0.3%).

Geographically, the majority of reports came from the United States (63,664; 96.5%), followed by the United Kingdom (1187; 1.8%), Japan (522; 0.8%), and other countries (601; 0.9%).

Report submissions increased significantly over time, with 4931 (7.5%) in 2022; 20,600 (31.2%) in 2023; 37,854 (57.4%) in 2024; and 2589 (3.9%) in the first quarter of 2025.

Table 3 shows a significant increase in the AE reports linked to tirzepatide from 2022 to the first quarter of 2025, particularly related to incorrect dose administration. The AE reports surged from 4931 (2022) to 37,854 (2024). Furthermore, the risk signals remained high, i.e., reporting odds ratio (ROR), as it consistently increased (22.15–27.31), peaking in 2023. Also, proportional reporting ratios (PRRs) were similarly high (16.80–19.87), indicating a strong association. However, EBGM and IC slightly declined in 2024 but still signaled significant risk.

### 3.1. Most Reported Adverse Events (Consistently Top 5)

Reviewing individual adverse events from 2022 to Q1 2025 showed that “Incorrect Dose Administered” was the most common, with 19,461 reports (Table 4). This event type saw a significant increase over the years, rising from 1248 in 2022 to 9800 in 2024, as well as adding 2589 more reports in Q1 2025. “Injection-site Pain” was the second most reported event, with 9849 reports. This adverse event saw a steady increase each year, reaching its peak at 5273 reports in 2024. Nausea ranked third, with 7678 reports from 2022 to Q1 2025. It remained a consistent gastrointestinal complaint, increasing from 655 in 2022 to 3602 in 2024. The fourth most reported event was “Off-label Use”, with 6993 total reports, which peaked in 2023 (3162 reports). The fifth was “Extra Dose Administered”, which accounted for 5044 reports. This event also showed a sharp increase from 283 in 2022 to 2984 reports in 2024.

### 3.2. Class-Effective AEs

Some AEs might be related to the class of the GLP-1/GIP agonists including decreased appetite and weight loss. Also, blood glucose fluctuations were reported in 2023–2024 and may reflect variability in glycemic control in some patients.

**Figure 1:**
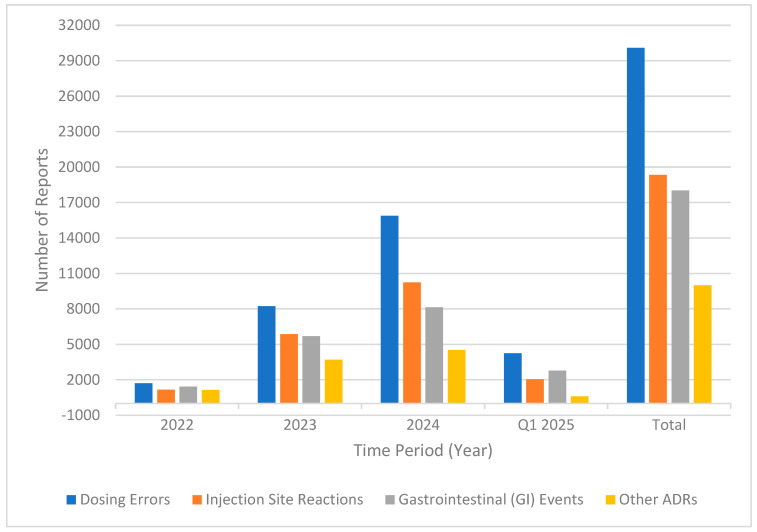
Trends in adverse event categories (2022—Q1 2025).

As part of our effort to categorize adverse events, we grouped them into the following four main categories: dosing errors, injection-site reactions, gastrointestinal (GI) events, and other adverse drug reactions (ADRs). The most common category was dosing errors, with a total of 30,075 reports during the study period. Dosing errors included incorrect doses, extra doses, accidental underdoses, missed doses, and improper schedules for product administration. There was a significant year-over-year increase in this category, rising from 1715 in 2022 to 15,887 in 2024 and 4241 in Q1 2025 (Figure 1).

Injection-site reactions included 19,334 reports, the second most common category. These reactions included injection-site pain, bleeding, redness, and bruising, with the most reports coming in at 10,245 in 2024.

Gastrointestinal (GI) events, including nausea, vomiting, diarrhea, constipation, decreased appetite, and abdominal pain, resulted in a total of 18,018 reports. These events increased consistently over the years, rising from 1423 in 2022 to 8133 in 2024, with 2771 already recorded in Q1 2025.

Other adverse reactions to tirzepatide included off-label use, fatigue, headache, weight loss, and increased blood sugar, totaling 9995 reports. Although this category was the least common overall, it experienced a steady increase from 1153 reports in 2022 to 4532 in 2024.

## 4. Discussion

This study reveals several critical insights into the safety profile and real-world use of tirzepatide. The data highlight a significant increase in reported ADEs from 2022 to 2025, with incorrect dose administration emerging as the most prominent issue. This trend aligns with the growing use of tirzepatide, particularly in the United States, which accounted for 96% of the country-specified reports. While there was a study that focused on the AE reports for tirzepatide in the U.S. FAERS database for the period of 2022–2023 [24], our study explored such reported AEs from 2022 through to the first quarter of 2025, providing a more comprehensive view covering the overall safety profile of this medication. The demographics indicate that females were the most frequent reporters, suggesting a potential sex difference (67% female), which aligns with the previous literature showing that females report more ADEs than males [25,26]. Given that tirzepatide is approved by the FDA for the management of type 2 diabetes and obesity, the predominance of reports originating from individuals aged 40–59 years is likely attributable to the high burden of these chronic conditions within this age group [27,28]. However, it is important to note that, as is the nature of the database, there are reports that do not include age information, which limits the detail of age-specific analyses—a common issue in spontaneous reporting systems. One notable trend was the high number of reports submitted by consumers, which accounted for 93.7%. This indicates that there is awareness among consumers/patients about the adverse events that they experienced or the errors that occurred during the usage of the products. Also, since these errors are related to administration, consumers are the most accurate population to describe these events. Moreover, we acknowledge the issue of underreporting by healthcare professionals, especially pharmacists and physicians. Therefore, there is a need to encourage more active reporting from physicians and pharmacists to improve the clinical validity of pharmacovigilance data and support earlier signal detection.

The high number of U.S.-origin reports (96.5%) indicates a regional imbalance that can skew global safety signal detection. This highlights the need for more balanced international participation in pharmacovigilance systems [20,21]. There was a significant increase in report submissions over time, jumping from 5462 in 2022 to 23,497 in 2023, 38,797 in 2024, and 9666 in the first quarter of 2025. The growth in AE reports from 2022 to 2024 could be due to a greater awareness of drug safety, more widespread use of newer medications like tirzepatide, and easier online reporting systems.

In previously published research, there were no reported dose-dependent ADEs [24]. However, the surge in ADEs reported in our study, especially incorrect dose administration, is concerning. The nearly 8-fold increase in such reports from 2022 to 2024 underscores the challenges in prescribing and administering tirzepatide. The consistently elevated risk signals, as evidenced by the high ROR and PRR, further emphasize the strong association between tirzepatide and dosing errors. Although the EBGM and IC showed slight declines in 2024, they remained significant, indicating ongoing safety concerns. The nature and frequency of dosing errors—such as double-dosing, missed doses, or incorrect titration—with tirzepatide mirror those documented for semaglutide and dulaglutide. These errors are primarily attributable to the following two class-wide features: the once-weekly administration schedule, which can cause patients to lose track of their injection day, and the mandatory dose-escalation protocols designed to improve gastrointestinal tolerability [29]. For instance, tirzepatide’s titration from 2.5 mg up to 15 mg is analogous in complexity to semaglutide’s escalation from 0.25 mg to 2.0 mg. There is no evidence to suggest that tirzepatide’s pre-filled pen device, which is similar to that of dulaglutide, or its formulation contributes to a higher rate of user error compared to other agents in the class [30]. Such safety concerns, especially for dose-dependent ADEs of tirzepatide, could lead to severe gastrointestinal AEs, as evident by related clinical trials [31]. These findings suggest a need for enhanced education and training for healthcare providers (e.g., prescribers, pharmacists, and nurses) and patients to mitigate dosing errors and improve medication safety. Such strategies could also mitigate injection-site issues and help such medication users. As the American Diabetes Association has issued practical guidelines for GLP-1 medications’ administration [32], it would be better for clinicians to update their knowledge and educate their patients based on these guidelines to minimize such ADEs.

Injection-site reactions were also among the top reported AEs, with injection-site pain particularly prevalent. These events are consistent with the known side effects of GLP-1/GIP agonists, the class to which tirzepatide belongs [33]. Injection-site reactions, such as erythema, pruritus, and swelling, were reported at a low and consistent frequency across the tirzepatide SURPASS clinical trials, occurring in approximately ≤1–2% of participants [34]. This incidence is directly comparable to the rates observed with other established GLP-1 RAs, including approximately 1% with semaglutide in the SUSTAIN program and 1–2% with dulaglutide in the AWARD trials [30]. These events are typically mild and transient in nature. Their similar prevalence across different molecules indicates they are a consequence of the subcutaneous administration of peptide-based therapies rather than a specific issue related to tirzepatide’s dual glucose-dependent insulinotropic polypeptide (GIP) and GLP-1 receptor agonism [29]. These events have persisted over the years, underscoring the importance of proactive management strategies. Providing patient counseling on injection techniques, along with standardized training and clear guidance, can help minimize discomfort, improve tolerability, and boost adherence in long-term injectable therapies.

The third major category was gastrointestinal (GI) adverse events. This is particularly relevant to tirzepatide, as clinical trials (e.g., SURPASS and SURMOUNT studies) and post-marketing surveillance have consistently reported nausea, vomiting, diarrhea, and constipation as the most frequent adverse effects [35]. These symptoms, although often transient, may lead to discontinuation of treatment or dose interruption [36]. A 2024 pharmacovigilance study noted a dose-dependent increase in GI adverse events among tirzepatide users, especially during the initiation and titration phases [37]. These GI adverse events are similar to GLP-1 agonists [33]. Therefore, such surveillance studies are crucial for identifying any major safety concerns with tirzepatide or similar medications as soon as possible. By proactively managing GI side effects through strategies such as dose adjustments, dietary changes, and anti-nausea medication, healthcare providers can help prevent patients treated with tirzepatide from having to discontinue treatment.

The other ADRs category, including fatigue, headache, weight changes, and blood glucose abnormalities, totaled 9995 reports in the EudraVigilance database [38]. The introduction of new AEs, such as blood glucose fluctuations from 2022 to 2024, suggests evolving patterns in tirzepatide use and associated risks [38]. The class-effective adverse events align with the pharmacodynamic effects of tirzepatide. However, the reports of blood glucose variability in some patients indicate the need for careful monitoring, particularly in individuals with diabetes, to ensure optimal glycemic control [35]. These results may reflect broader trends in prescribing practices or challenges in patient adherence and warrant further investigation. Regulatory bodies and institutions should track off-label prescribing patterns, particularly with newer agents like tirzepatide, to ensure appropriate patient selection and risk–benefit analysis.

### Limitations

Like most spontaneous reporting systems, this study has several limitations, including underreporting, a lack of denominator data, and missing clinical details, which impact the interpretation of the findings. A large number of reports were missing key demographic and clinical information, as follows: age was unknown in 35.9%, sex was not reported in 12.8%, and clinical outcomes were missing in 89.7% of reports. These gaps may limit the ability to conduct in-depth analyses, estimate incidence rates, or draw firm conclusions about causality and severity. Furthermore, the data show a strong geographic concentration, with 96.5% of reports coming from the United States. This regional bias may limit the applicability of the findings and could mask potential safety signals in other populations or healthcare systems. Additionally, causality cannot be deduced from the FAERS data, and duplicated or incomplete reports might affect signal strength. Due to the spontaneous nature of the FAERS reports, the causality between tirzepatide and the reported adverse events cannot be established. The data reflect associations that may suggest a potential safety signal but do not confirm direct cause–effect relationships. Therefore, future research might be needed to explore the underlying causes of these AEs and evaluate the effectiveness of mitigation strategies in real-world settings. Furthermore, qualitative research investigating the underlying reasons and factors that may lead to such safety concerns from users’ and healthcare providers’ perspectives may be essential to reduce these concerns for these medications or similar ones.

## 5. Conclusions

Tirzepatide’s increasing use in the management of diabetes and obesity is reflected in emerging pharmacovigilance trends. The findings underscore the importance of vigilant monitoring and targeted interventions to address the safety concerns associated with tirzepatide, particularly dosing errors and injection-site reactions. Healthcare providers should prioritize patient education, clear dosing instructions, and regular follow-ups to minimize risks.

## Figures and Tables

**Table 1:** Contingency table for disproportionality analyses.

	Adverse Drug Reaction of Interest	Other Adverse Drug Reactions
Drug of interest	a	b
All other drugs in the database	c	d

**Table 2:** Demographics of tirzepatide adverse event reports.

Characteristic	Category	Number of Reports (%)
**Sex**	Female (F)	44,120 (66.9)
	Male (M)	13,410 (20.3)
	Unknown (UNK)	8444 (12.8)
**Age**	(<18 years)	16 (0.02)
	(18–39 years)	7519 (11.4)
	(40–59 years)	21,809 (33.1)
	(60–89 years)	12,943 (19.6)
	(≥90 years)	21 (0.04)
	Unknown (UNK)	23,666 (35.9)
**Reporter** **Type**	Healthcare Professional (HP)	2062 (3.1)
	Medical Doctor (MD)	1110 (1.7)
	Pharmacist (PH)	691 (1.0)
	Consumer (CN)	61,838 (93.7)
	Lawyer (LW)	222 (0.3)
	Other/Unknown	51 (0.1)
**Outcome**	Death (DE)	221 (0.3)
	Hospitalization (HO)	2418 (3.7)
	Disability (DS)	181 (0.3)
	Other (OT)	3983 (6.0)
	Unknown (UNK)	59,171 (89.7)
**Reporting Country**	United States (US)	63,664 (96.5)
	United Kingdom (GB)	1187 (1.8)
	Japan (JP)	522 (0.8)
	Others (20+ countries)	601 (0.9)
**Reporting Year**	2022	4931 (7.5)
	2023	20,600 (31.2)
	2024	37,854 (57.4)
	Q1 2025	2589 (3.9)

**Table 3:** Risk of incorrect dose administered and the use of tirzepatide during 2022 to Q1 2025.

Year	Number of Incorrect Dose Administration ADEs	Reported ADEs for the Drug of Interest	ROR (95% CI)	PRR (95% CI)	EBGM	IC
**2022**	1248	4931	22.15 (20.75–23.65)	16.80 (15.74–17.93)	16.01	4.00
**2023**	5824	20,600	27.31 (26.43–28.23)	19.87 (19.23–20.54)	16.10	4.00
**2024**	9800	37,854	23.43 (22.82–24.05)	17.62 (17.16–18.09)	12.68	3.66
**Q1 2025**	2589	11,206	17.61 (16.75–18.52)	13.78 (13.10–14.49)	10.13	3.34

**Table 4:** The most frequent adverse event signals associated with tirzepatide between 2022 and Q1 2025.

Adverse Event	2022	2023	2024	Q1 2025	Total
Incorrect dose administered	1248	5824	9800	2589	19,461
Injection-site pain	592	2946	5273	1038	9849
Nausea	655	2390	3602	1031	7678
Off-label use	748	3162	2704	379	6993
Extra dose administered	283	1078	2984	699	5044
Injection-site hemorrhage	289	1344	2056	370	4059
Diarrhea	286	1056	1787	619	3748
Injection-site erythema	153	987	1747	376	3263
Vomiting	268	885	1573	515	3241
Accidental underdose	—	—	1981	485	2466
Constipation	—	703	1171	370	2244
Product dose omission issue	—	593	1122	468	2183
Injection-site bruising	137	588	1169	269	2163
Blood glucose increased	—	547	1019	—	1566
Fatigue	111	—	809	222	1142
Inappropriate schedule of product administration	184	737	—	—	921
Decreased appetite	214	657	—	—	871
Abdominal pain upper	—	—	—	236	236
Weight decreased	192	—	—	—	192
Headache	102	—	—	—	102

## Data Availability

The FDA Adverse Event Reporting System (FAERS) quarterly data extract files are publicly available and can be obtained at the following: https://fis.fda.gov/extensions/FPD-QDE-FAERS/FPD-QDE-FAERS.html (accessed on 5 April 2025).
